# Kinetic Analysis of the Curing of a Partially Biobased Epoxy Resin Using Dynamic Differential Scanning Calorimetry

**DOI:** 10.3390/polym11030391

**Published:** 2019-02-27

**Authors:** Diego Lascano, Luis Quiles-Carrillo, Rafael Balart, Teodomiro Boronat, Nestor Montanes

**Affiliations:** 1 Escuela Politécnica Nacional, Quito 17-01-2759, Ecuador; dielas@epsa.upv.es; 2 Technological Institute of Materials (ITM), Universitat Politècnica de València (UPV), Plaza Ferrándiz y Carbonell 1, 03801 Alcoy, Spain; rbalart@mcm.upv.es (R.B.); tboronat@dimm.upv.es (T.B.); nesmonmu@upvnet.upv.es (N.M.)

**Keywords:** cure kinetics, epoxy resin, bio-sourced epoxy resin, differential scanning calorimetry (DSC)

## Abstract

This research presents a cure kinetics study of an epoxy system consisting of a partially bio-sourced resin based on diglycidyl ether of bisphenol A (DGEBA) with amine hardener and a biobased reactive diluent from plants representing 31 wt %. The kinetic study has been carried out using differential scanning calorimetry (DSC) under non-isothermal conditions at different heating rates. Integral and derivative isoconversional methods or model free kinetics (MFK) have been applied to the experimental data in order to evaluate the apparent activation energy, *E_a_*, followed by the application of the appropriate reaction model. The bio-sourced system showed activation energy that is independent of the extent of conversion, with *E_a_* values between 57 and 62 kJ·mol^−1^, corresponding to typical activation energies of conventional epoxy resins. The reaction model was studied by comparing the calculated *y*(*α*) and *z*(*α*) functions with standard master plot curves. A two-parameter autocatalytic kinetic model of Šesták–Berggren [SB(*m*,*n*)] was assessed as the most suitable reaction model to describe the curing kinetics of the epoxy resins studied since it showed an excellent agreement with the experimental data.

## 1. Introduction

Epoxy resins are low molecular weight pre-polymers with at least one epoxide group in their structure. These epoxy rings are usually located in terminal positions due to the high reactivity of this position [1]. Currently epoxy resins are the most used thermosetting resins due to the huge range of properties they can cover such as tensile strength, high adhesion strength, low shrinkage, good chemical resistance, and low volatile emission, among others [2,3,4,5]. These outstanding properties make these resins suitable for high technological sectors such as the automotive and aerospace industries. In addition, their versatility makes them useful for industrial applications such as adhesives, paints, surface coatings, advanced composite materials, electrical and electronic components, and high-performance membranes for separation and filtration, among others [3,6,7,8,9,10,11,12,13,14]. The final properties of a structural epoxy system are greatly influenced by several factors, which include the chemical structure of both the epoxy resin and the curing agent and external factors related to the curing procedure.

Despite the huge range of epoxy resins available worldwide, undoubtedly the most used resin is that based on diglycidyl ether of bisphenol A (DGEBA), which is obtained by reacting bisphenol A (BPA), and epichlorohydrin [15]. Although some epoxy resins can crosslink via homopolymerization [16], the use of hardeners is usually mandatory to crosslink the liquid epoxy resin. There is a wide variety of commercial hardeners for epoxy systems, with amine systems being one of the most employed. Amines can crosslink an epoxy resin by directly cross-linking or by catalytic cross-linking mechanisms [17,18]. These amine hardeners could be both primary and secondary, which act as reactive hardeners, or tertiary amines, which act as catalytic agents. The crosslinking structure are obtained by the reaction between the epoxide group and the hydrogens contained in the amine groups [19]. One of the main features of amine hardeners is that they can provide curing at low temperatures, or even at room temperature [20,21], which is a key factor compared to other hardeners, such as carboxylic acids or anhydrides, that typically need high temperatures to start and complete the cross-linking process [22,23,24,25].

Epoxy resins are petroleum-derived materials that have a remarkable impact on the carbon footprint. This fact, in conjunction with the increasing sensitivity of our society about the environment and sustainable development, are the leading forces toward the development of new environmentally friendly resins that are totally or partially bioderived [26,27,28,29]. One of the most promising sources for industrial epoxy resins are vegetable oils. These consist of a triglyceride structure in which three different fatty acids are chemically attached to glycerol by ester bonds. Unsaturated fatty acids, such as oleic, linoleic, and linolenic acids, with one, two, and three unsaturations, respectively, are the most interesting fatty acids as their unsaturations can be easily converted into oxirane rings via epoxidation [30]. In addition, the metathesis of double bonds has been proposed as a process for undergoing epoxidation of unsaturated fatty acids, among others [31,32]. The most common oils used for epoxidation are canola, corn, palm, linseed, soybean, castor, and so on [33]. Nevertheless, from an industrial point of view, epoxidized soybean oil (ESO) and epoxidized linseed oil (ELO) are, the most used plant-derived epoxy resins due to an excellent combination of properties. Moreover, they offer a competitive cost since they are widely used in the poly(vinyl chloride) (PVC) industry as secondary plasticizers [34,35,36,37]. In addition to this industrial use, epoxidized vegetable oils can be considered epoxy resins, and therefore, they can be cross-linked with the appropriate hardener. Samper et al. [38] obtained eutectic mixtures of epoxidized soybean oil (ESO) and epoxidized linseed oil (ELO) cured with maleic anhydride. Park et al. [39] focused their research on the effect of the epoxidized castor oil (ECO) in blends with DGEBA-based epoxy resins. Pawar et al. [40] formulated a fully bio-based resin, based on blends of epoxidized cottonseed oil (ECO) and epoxidized algae oil (EAO).

As indicated previously, the final properties are highly influenced by the curing conditions in terms of the time–temperature cycle. At the industrial scale, short curing times are preferred and this leads to the need of higher curing temperatures (more aggressive curing cycles). These aggressive conditions also provide internal stresses due to the fastness of the crosslinking process, which in turn, leads to higher *T_g_* values.

During the crosslinking process between a base epoxy resin and the corresponding hardener, an exothermic reaction occurs. Due to the exothermicity, the temperature of the resin increases, and subsequently, the crosslinking rate raises up in an autocatalytic process. In fact, it is important to control the released heat as in some times it can degrade (even burn) the resin leading to a counterproductive effect on final properties [41]. In order to obtain a cured thermosetting with optimal properties, the knowledge of the kinetics is essential [42]. The aim of this research is to study the curing kinetics of a partially biobased epoxy resin using dynamic differential scanning calorimetry (DSC). The work is focused on obtaining the kinetic triplet: *E_a_*, *A*, and f(α). *E_a_* is obtained by isoconversional methods or model free kinetics (MFK), both differential and integral and the reaction model is obtained by comparison of the y(α) and z(α) characteristic curves with standard master plots.

## 2. Theoretical Background 

Most of the thermally activated processes are based on the general kinetic expression, as shown in Equation (1), where the conversion rate $\frac{d\alpha }{dt}$ is a function of *T* and α [43]:

(1) $$\frac{d\alpha }{dt}=K\left(T\right)f\left(\alpha \right)$$

where *K*(*T*) is the rate coefficient, which depends on temperature *T*, and f(α) represents the reaction model, which takes different forms depending on the mechanism of the process. The temperature dependence of *K*(*T*) could be parameterized through the Arrhenius expression shown in Equation (2):

(2) $$K\left(T\right)=A\;e^{\frac{-E_{a}}{RT}}$$

where *A* is the pre-exponential factor, *E_a_* is the apparent activation energy, *R* refers to the universal gas constant, and *T* is the absolute temperature. By substituting the Arrhenius temperature coefficient, Equation (1) can be written as Equation (3), which represents the general rate equation for the kinetic study. As one can realize, this is a time-dependent equation, and subsequently, it can be applied to any curing cycle.

(3) $$\frac{d\alpha }{dt}=A\;e^{\frac{-E_{a}}{RT}}\;f(\alpha )$$

This time-dependent expression can be converted into a temperature domain by considering Equation (4), which shows the heating rate β definition:

(4) $$\beta =\frac{dT}{dt}$$

By substituting Equation (4) into Equation (3), we obtain the general equation for kinetic analysis for dynamic heating cycles:

(5) $$\beta \frac{d\alpha }{dT}=A\;e^{\frac{-E_{a}}{RT}}f(\alpha )$$

### Isoconversional Methods: Model-Free Kinetics (MFK)

Isoconversional methods are derived from the basic isoconversional principle that assumes that the reaction rate at a constant conversion ($\alpha_{c}$) depends only on temperature. If we take natural logarithms on both sides of Equation (1), we obtain:

(6) $$\ln\frac{d\alpha }{dt}=\ln\;K(T)+\ln\;f(\alpha )$$

Then, taking partial derivatives with respect to the inverse temperature (1/*T*) on Equation (6) at a constant α = $\alpha_{i}$, Equation (7) is derived:

(7) $${\left[\frac{\partial \ln\left(\frac{d\alpha }{dt}\right)}{\partial \;T^{-1}}\right]}_{\alpha_{i}}={\left[\frac{\partial \ln K(T)}{\partial \;T^{-1}}\right]}_{\alpha_{i}}+{\left[\frac{\partial \ln f(\alpha )}{\partial \;T^{-1}}\right]}_{\alpha_{i}}$$

Isoconversional conditions, meaning that it has a constant α value of $\alpha_{i}$ such that its corresponding f(α) value is also constant. Hence, the second term on the right-hand side of Equation (7) is zero. The first term on the right-hand side of Equation (7) can be obtained by taking the partial derivative of the logarithmic Arrhenius coefficient in Equation (2) with respect to 1/*T*, as shown in Equation (8):

(8) $${\left[\frac{\partial \ln K(T)}{\partial \;T^{-1}}\right]}_{\alpha_{i}}={\left[\frac{\partial \ln A\;e^{\frac{-E_{a}}{RT}}}{\partial \;T^{-1}}\right]}_{\alpha_{i}}=\frac{-E_{a,\alpha_{i}}}{R}$$

By combining Equations (7) and (8), the isoconversional methods indicate that it is possible to obtain *E_a_* (at a particular $\alpha_{i}$ = constant) without assuming any reaction model, as shown in Equation (9). For this reason, sometimes these methods are called model-free kinetic methods (MFK).

(9) $${\left[\frac{\partial \ln\left(\frac{d\alpha }{dt}\right)}{\partial \;T^{-1}}\right]}_{\alpha_{i}}=\frac{-E_{a,\alpha_{i}}}{R}$$

Isoconversional methods can give accurate values of the apparent activation energy *E_a_*. These methods can be classified as differential, such as Friedman’s method, or integral methods, such as the Flynn–Wall–Ozawa (FWO), Kissinger–Akahira–Sunose (KAS), and Starink methods. The Friedman method [44] is one of the most common differential isoconversional methods used to evaluate the apparent activation energy. By assuming that the reaction model f(α) does not change with the conversion α, the basic differential expression (Equation (3)) can be written in a natural logarithm form, as shown in Equation (10):

(10) $$\ln{\left(\frac{d\alpha }{dt}\right)}_{\alpha ,i}=\ln\left[A\;f(\alpha )\right]-\frac{E_{a}}{{RT}_{\alpha ,i}}=\ln{\left(\beta \frac{d\alpha }{dT}\right)}_{\alpha ,i}$$

As suggested by Equation (10), by plotting $\ln{\left(\beta \frac{d\alpha }{dT}\right)}_{\alpha ,i}$ versus $\frac{1}{T_{\alpha ,i}}$, the *E_a_* can be calculated through the slope of the linear fitting. This method must be evaluated for each value of the conversion α. As suggested by Sergey Vyazovkin et al. [43], to check its possible variation with the conversion α, it is recommended to start from $\alpha_{i}$ = 0.05 up to $\alpha_{i}$ = 0.95 with an increasing step size of 0.05. The Friedman method does not make any other assumption, and in a first approach, it gives a quite accurate value of *E_a_*.

Isoconversional integral methods are based on integration of Equation (3) or Equation (5), as observed in Equation (11):

(11) $$\int_{0}^{\alpha }\frac{d\alpha }{f(\alpha )}=\frac{A}{\beta }\int_{T_{0}}^{T}e^{\frac{{-E}_{a}}{RT}}\;dT=A\int_{0}^{t}e^{\frac{{-E}_{a}}{RT}}\;dt$$

The integral of the temperature-dependent expression from the Arrhenius constant is the so-called “temperature integral” and does not have a direct analytical solution. For this reason, different approximations have been proposed and used, leading to different methods, and obviously, to different accuracies on the *E_a_* estimation. The Flynn–Wall–Ozawa (FWO) method shows very low accuracy as it uses a rude approximation of the temperature integral. Despite this, the FWO is one of the most commonly used. It follows Equation (12) [45]. This suggests that a plot of $\ln\left(\beta_{i}\right)$ versus the inverse temperature (1/*T_α_*) for a constant conversion α gives a straight line whose slope is −1.052/*R*.

(12) $$\ln(\beta_{i})=\mathrm{constant}-1.052\frac{E_{a}}{{RT}_{\alpha }}$$

The Kissinger–Akahira–Sunose [46] method gives more accurate *E_a_* values as it uses a more accurate solution of the temperature integral. The basic expression for the KAS method is shown in Equation (13), which suggests a linear correlation between $\ln\left(\frac{\beta_{i}}{T_{\alpha ,i}^{2}}\right)$ and the inverse temperature (1/*T_α_*) for a particular conversion α value.

(13) $$\ln\left(\frac{\beta_{i}}{T_{\alpha ,i}^{2}}\right)=\mathrm{constant}-\frac{E_{a}}{{RT}_{\alpha }}$$

A better evaluation of the activation energy (*E_a_*) can be done using the Starink expression, which is similar to that of the KAS method, but the particular parameters are optimized, as observed in Equation (14). In a similar way to the above-mentioned methods, a plot of $\ln\left(\frac{\beta_{i}}{T_{\alpha ,i}^{1.92}}\right)$ against (1/*T_α_*) for a particular conversion α value gives a linear correlation whose slope is −1.0008/*R* [47].

(14) $$\ln\left(\frac{\beta_{i}}{T_{\alpha ,i}^{1.92}}\right)=\mathrm{constant}-1.0008\frac{E_{a}}{{RT}_{\alpha }}$$

Although it is not considered an isoconversional method, the Kissinger method (not to be confused with the Kissinger–Akahira–Sunose method) is based on the peak temperature (*T_p_*) observed for the conversion rate at different heating rates $\beta_{i}$ [48]. This method is widely used because of its simplicity. It assumes that $\frac{d}{dt}\left(\frac{d\alpha }{dt}\right)=0$ at the temperature for the maximum conversion rate (*T_p_*). The assumptions of the Kissinger method are only applicable if the conversion for this peak temperature α_m_ is almost constant for a series of heating rates, $\beta_{i}$. The Kissinger method follows Equation (15). One important drawback of the Kissinger method is that it only provides a single *E_a_* value (at α_m_) and cannot follow the evolution of *E_a_* with the conversion α. Despite this, it is widely used due to its simplicity.

(15) $$\ln\left(\frac{\beta }{T_{p}^{2}}\right)=\ln\left(\frac{AR}{E_{a}}\right)-\frac{E_{a}}{{RT}_{p}}$$

By plotting $\ln\left(\frac{\beta }{T_{p}^{2}}\right)$ versus $\frac{1}{T_{p}}$, the activation energy can be obtained through the slope of the linear fit. One of the restrictions of the Kissinger method is that it assumes a single-step process. To check the existence of a single-step degradation process, different methods have been proposed. In particular, it is worthy to note the methodology described by Farjas et al. [49]. This method is not only based on the peak temperature, but also on the peak width measured as the time corresponding to the full width at half maximum (Δ*t_FWHM_*), which is more sensitive to the existence of multiple processes. If a plot of the Δ*t_FWHM_* against 1/*T_p_* gives a linear correlation, and the obtained *E_a_* by linear fitting is similar to that obtained by the Kissinger method, then it is possible to conclude that the curing process takes place in a single step (despite the complexity of the occurring reactions).

Isoconversional methods are very useful for giving an accurate estimation of the *E_a_* and do not assume any reaction model f(α). Polymerization processes are very complex, as indicated by D.R. D’hooge et al. [50]. In particular they use multi-scale modelling to unveil some of these complex processes governed by free radical polymerization (FRP) or by controlled radical polymerization (CRP). They also pay special attention to chain mobility, which is restricted by crosslinking. To obtain the kinetic triplet, it is necessary to determine the reaction model. One of the most suitable methods is the use of the Málek method [51], which allows determining the reaction model by experimentally calculating two functions defined as y(α) and z(α), as indicated in Equations (16) and (17), with *x* = *E_a_*/*RT*, i.e., the reduced temperature:

(16) $$y(\alpha )=\frac{d\alpha }{dt}e^{x}$$

(17) $$z(\alpha )=\pi (x)\frac{d\alpha }{dt}\frac{T}{\beta }$$

The particularity of these two functions is the following. Regarding y(α), if we substitute Equation (3) into Equation (16), then Equation (18) is obtained. The relevance of this function is that it can be calculated numerically using Equation (16), and as suggested by Equation (18), it provides a plot representation proportional to f(α) by the factor *A*. Therefore, y(α) gives a clear idea of the reaction model.

(18) y(α)=A f(α)

Regarding the z(α) function, the solution to the integral expression (Equation (11)) can be defined as:

(19) $$g(\alpha )=\frac{{AE}_{a}}{\beta R}\;P(x)$$

where P(x) represents a particular approximation of the temperature integral. The relation between P(x) and π(x) is shown in Equation (20):

(20) $$P(x)=\frac{e^{-x}}{x}\;\pi (x)$$

By combining Equations (3), (19), and (20), Equation (17) results in the following expression:

(21) z(α)=f(α) g(α)

which suggests that the calculated z(α) using Equation (17), can be compared with different theoretical z(α) curves with well known f(α) and g(α) functions as shown in Table 1.

Senum and Yang have proposed different approximations of π(x) in a rational form [52] and Perez-Maqueda et al. [53] have indicated that the use of a rational approximation of π(x) with a fourth degree polynomial is enough to give good accuracy. Flynn [54] suggested a correction for the fourth rational approximation expression, as we can see at the Equation (14). These functions y(α) and z(α) can be calculated from experimental data.

(22) $$\pi (x)=\frac{x^{3}+18x^{2}+86x+96}{x^{4}+20x^{3}+120x^{2}+240x+120}$$

## 3. Experimental

### 3.1. Materials

The commercial epoxy resin used was a Resoltech^®^ 1070 ECO and the hardener was an amine-based type Resoltech^®^ 1074 ECO, both of them supplied by Castro Composites (Pontevedra, Spain). This partially bio-sourced epoxy resin is based on a mixture of a diglycidyl ether of bisphenol A (DGEBA) and a plant-based epoxy reactive diluent. As indicated by the supplier, the cured resin contains 31% biobased content (according to ASTM D6866-12) and maintains good transparency. The eco-epoxy resin and the hardener were mixed under the stoichiometric ratio 100:35 (%wt epoxy:%wt hardener), following the manufacturer’s recommendations.

**Scheme 1:**
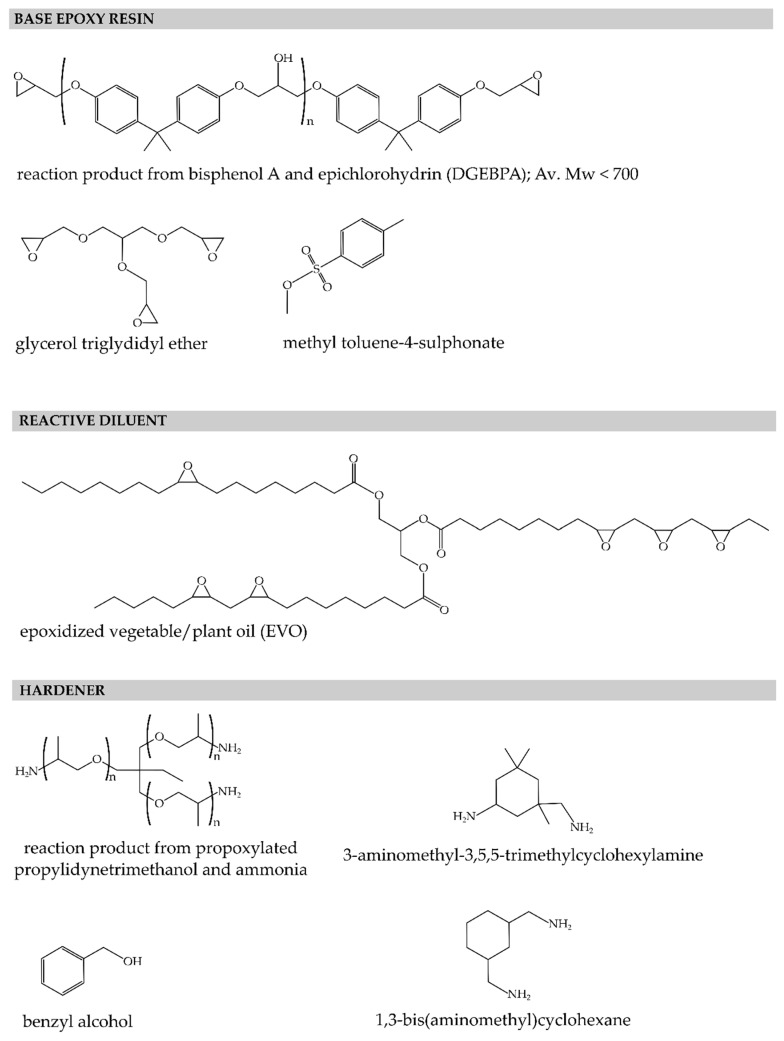
Schematic plot of the main chemical components of the partially bio-based epoxy resin Resoltech^®^ 1070 and its hardener, Resoltech^®^ 1074.

Scheme 1 shows the chemical structures of the components of the epoxy resin as well as the hardener. 

The main component on the epoxy resin is the reaction product of bisphenol A and epichlorohydrin, leading to a typical diglycidyl ether of bisphenol A (DGEBA) resin. With regard to the reactive diluent, it is mainly derived from plant or vegetable oils, in particular, epoxidized vegetable oils. Finally, the hardener is an amine type, mainly composed of 3-aminomethyl-3,5,5-trimethylcyclohexylamine and the reaction product of propoxylated propylidynetrimethanol and ammonia.

A preliminary FTIR characterization was carried out. The spectra were recorded using a spectrometer Spectrum BX from Perkin-Elmer (Madrid, Spain) together with a special holder for liquid samples. Data were collected from 20 scans between 5500 and 450 cm^−1^ at a spectral resolution of 4 cm^−1^. 

**Figure 1:**
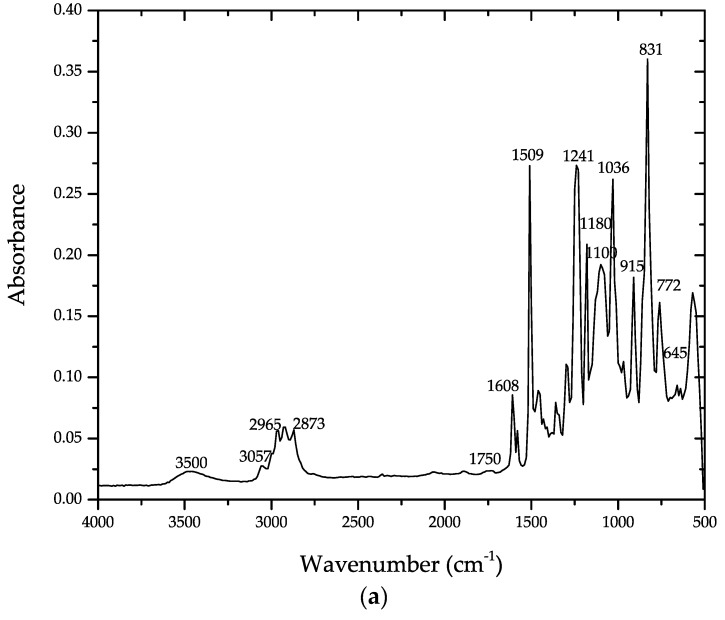

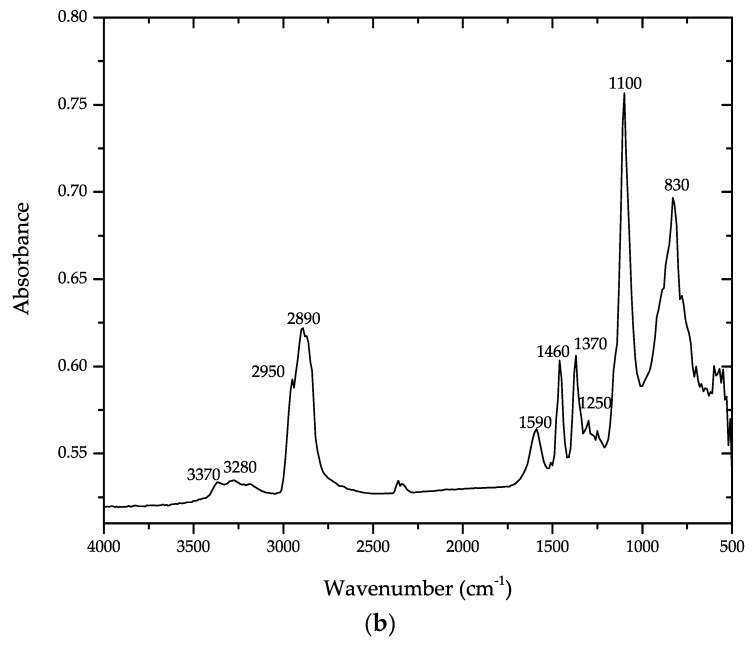
FTIR spectra of (**a**) base epoxy resin Resoltech^®^ 1070 based on DGEBPA, and (**b**) primary amine-based hardener Resoltech^®^ 1074.

Figure 1 shows the FTIR spectra of both the epoxy resin (Figure 1a) and the hardener (Figure 1b). The FTIR spectrum of the epoxy base epoxy resin shows the typical peaks and bands of a DGEBPA resin. These peak/band assignments are as follows: O–H stretching at about 3500 cm^−1^; stretching of the C–H of the oxirane ring at 3057 cm^−1^; stretching of C–H of CH_2_ and CH, both aromatic and aliphatic, at 2965–2873 cm^−1^; stretching of C=C of aromatic rings at 1608 cm^−1^; stretching of the C–C of aromatic rings at 1509 cm^−1^; stretching of the C–O–C of ethers at 1036 cm^−1^; asymmetric stretching of the C–O in oxirane rings at 1241 cm^−1^; stretching of the C–O of oxirane ring at 915 cm^−1^; stretching of the C–O–C of oxirane ring at 831 cm^−1^; and rocking of CH_2_ at 772 cm^−1^. 

With regard to the reactive diluent, an epoxidized vegetable oil contained overlapping peaks and bands of CH_2_, CH, and oxirane rings, while the ester groups typical of the triglyceride appeared with a weak signal as it was not the main component. These ester peaks were characterized by a weak signal at 645 cm^−1^ attributable to the O–C=O bending; a peak at 1180 cm^−1^, which overlaps with ester groups and corresponds to the antisymmetric stretch of C–O–C; and a weak signal at 1765–1720 cm^−1^, which is characteristic of the C=O stretch. These weak signals typical of the ester groups indicated the presence of triglycerides as reactive diluents. 

Regarding the hardener, it was an amine type (Figure 1b). The peak/band assignment was the following: NH stretching of –NH_2_ at 3370–3280 cm^−1^, symmetric and antisymmetric stretching of CH in –CH_2_ at 2950–2890 cm^−1^, symmetric and antisymmetric stretching of CH in –CH_2_–, –NH_2_ deformation at 1590 cm^−1^, scissors vibration of –CH_2_– at 1370 cm^−1^, symmetric deformation of –CH_3_ at 1370 cm^−1^, stretching of C–O–C ether groups at 1250 cm^−1^, stretching of –NH in C–NH_2_ primary amines located at 1100 cm^−1^, and wagging of –NH in R–NH_2_ primary amines.

Complementary to this preliminary characterization of the epoxy system, some additional properties are described. Some physical properties of the base epoxy, the hardener, and the uncured mixture are summarized in Table 2.

With regard to the reactivity of this system, it is worthy to note that the exothermic peak of the epoxy system during curing at 23 °C had a value of 185 °C and occurred at 31 min. It showed a gel time of 28 min when the mixture did not exceed a volume of 70 mL, as indicated by the supplier.

**Figure 2:**
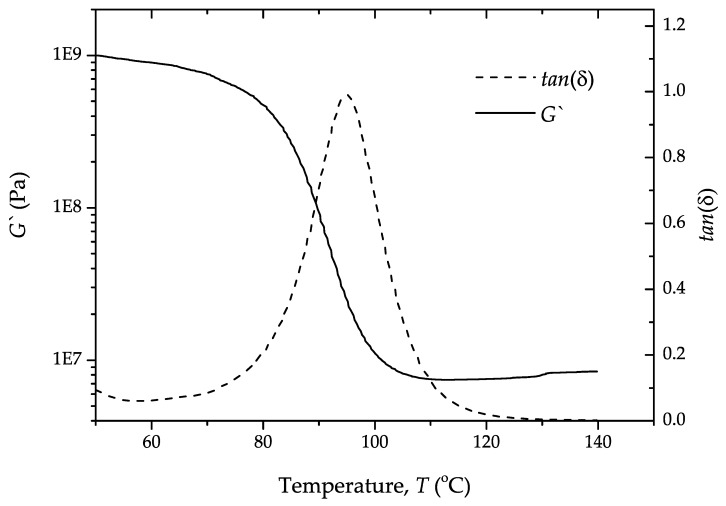
Plot evolution of the storage modulus (*G*′) and the damping factor (*tan* *δ*) of a partially bio-based epoxy resin after curing at 90 °C for 1 h and a post-curing cycle at 150 °C for 0.5 h.

In addition to these properties, the glass transition temperature, *T_g_*, of the cured resin was obtained via dynamic mechanical thermal analysis (DMTA). The curing cycle was 1 h at 90 °C and had a post-curing stage of 30 min at 150 °C. DMTA was carried out in an oscillatory rheometer AR-G2 supplied from TA Instruments (New Castle, DE, USA), using a special clamp system made for solid samples (4 × 10 × 40 mm^3^) working in torsion–shear conditions. Cured samples were subjected to a temperature sweep from 30 °C to 140 °C at a heating rate of 2 K min^−1^, with a maximum deformation (γ) of 0.1%, and a frequency of 1 Hz. Figure 2 shows the plot evolution of the storage modulus (*G*′) and the damping factor (*tan* *δ*). By taking the *T_g_* as the peak maximum of *tan* *δ*, a value of 95.3 °C was obtained for this cured epoxy system.

Finally, the Shore D hardness of the cured epoxy material was obtained in a 676-D durometer from J. Bot Instruments (Barcelona, Spain) at room temperature following ISO 868 standard, resulting in a Shore D value of 83.4 ± 1.7.

### 3.2. Differential Scanning Calorimetry (DSC) Kinetic Measurements

The curing kinetics of the eco-epoxy system was analyzed using differential scanning calorimetry (DSC) in a 821 DSC calorimeter from Mettler-Toledo, Inc. (Schwerzenbach, Switzerland). The sample weight was between 10–13 mg, following the recommendations of Vyazovkin et al. [55], suggesting that the sample’s mass should be an inverse proportion to the heating rate. The mixtures were prepared at room temperature using the stoichiometric ratio, and then the liquid mixture was stirred until homogenization. Subsequently, the corresponding mass was placed into a standard aluminium pan (40 μL) and sealed with a press. Finally, the samples were subjected to the following thermal program: first, an isothermal stage at 20 °C for 1 min was programmed to stabilize the sample temperature, then a heating process up to (250 °C–280 °C) at different heating rates, β (2.5, 5, 10, and 20 K min^−1^. This was followed by a cooling step down to 0 °C at a cooling rate of −20 K min^−1^, and finally, a second heating step up to 180 °C at 10 K min^−1^ was scheduled. All tests were carried out under a dry atmosphere with a constant nitrogen flow of 30 mL min^−1^. DSC was used to obtain the heat flow as a function of temperature (from the first heating), and the glass temperature (*T_g_*) (from the second heating).

Processing the thermal data from DSC is necessary to obtain the extent of conversion *α*. It could be expressed as indicated in Equation (23), where $\Delta H_{t}$ is the released enthalpy or heat at a particular time *t*, and $\Delta H_{T}$ is the total enthalpy released during the complete reaction. Equation (24) shows how the conversion rate can be obtained from the heat flow of the DSC thermograms.

(23) $$\alpha =\frac{\Delta H_{t}}{\Delta H_{T}}$$

(24) $$\frac{d\alpha }{dt}=\frac{1}{H_{T}}\cdot \frac{dH(t)}{dt}$$

## 4. Results and Discussion

### Estimation of the Apparent Activation Energy, E_a_

The curing reaction of the partially bio-based epoxy resin at different heating rates was studied using DSC. The first heating cycle was useful for determining the total enthalpy ($\Delta H_{T}$) and the temperature for the maximum curing rate (*T_p_*), while the second heating cycle was used to study the glass transition temperature (*T_g_*) of the cured resin. These values are gathered in Table 3 for different heating rates between 2.5 and 20 K min^−1^. It is important to remark that the *T_g_* decreased with increasing heating rate from 95.8 °C (2.5 K min^−1^) down to 91.1 °C (for 20 K min^−1^) which was also in accordance with the curing enthalpy ($\Delta H_{T}$). Low curing rates allow diffusion and crosslinking reactions to occur more readily. At high curing rates, the overall speed is high and does not allow free diffusion as a crosslinked structure is immediately formed [56].

**Figure 3:**
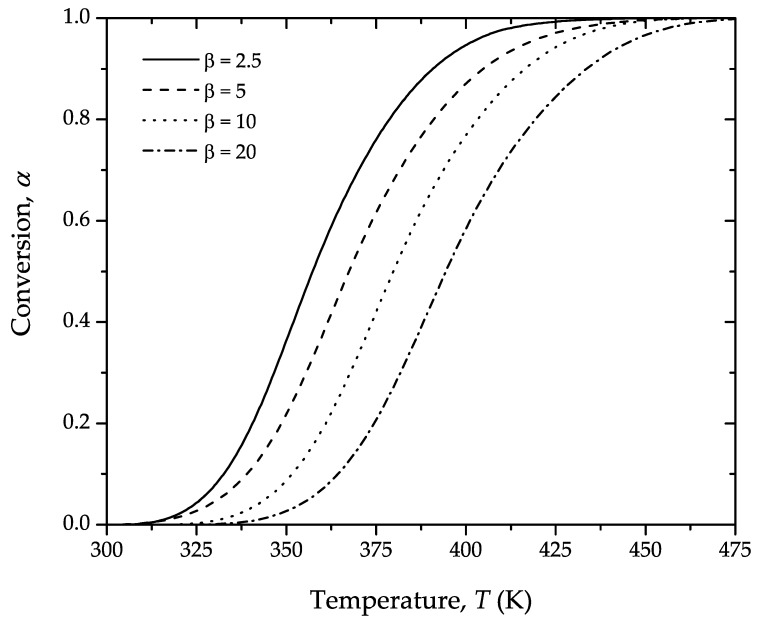
Fractional conversion of the curing of a partially bio-based epoxy resin as a function of temperature for different heating rates, β.

Figure 3 shows the conversion α as a function of the absolute temperature *T*. As the heating rate β increased, the characteristic sigmoidal curve moved toward higher values.

**Figure 4:**
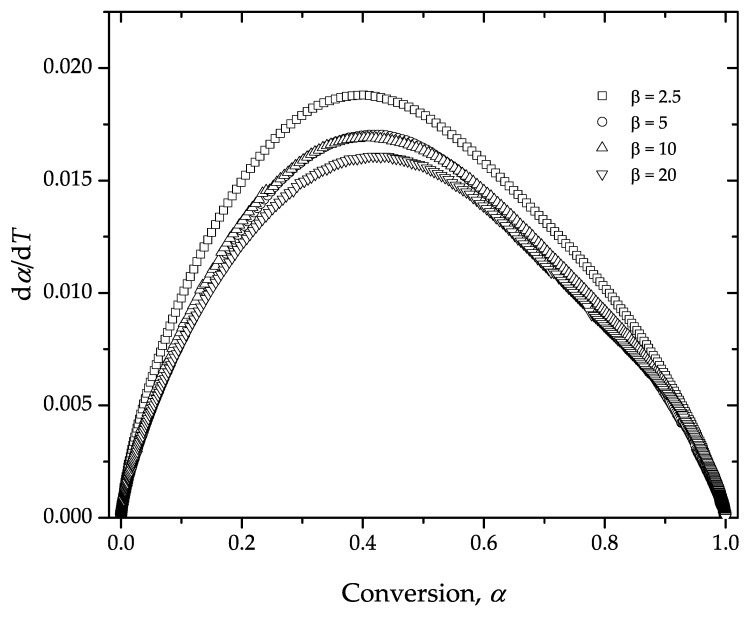
The curing rate corresponding to the crosslinking of a partially bio-based epoxy resin as a function of the conversion, *α*, for different heating rates, β.

Figure 4 shows a plot of the curing rate ($\frac{d\alpha }{dT}$) as a function of the conversion *α* obtained using Equation (24). The geometry of these plots is almost the same, thus suggesting there is no change in the reaction model with the heating rate.

**Figure 5:**
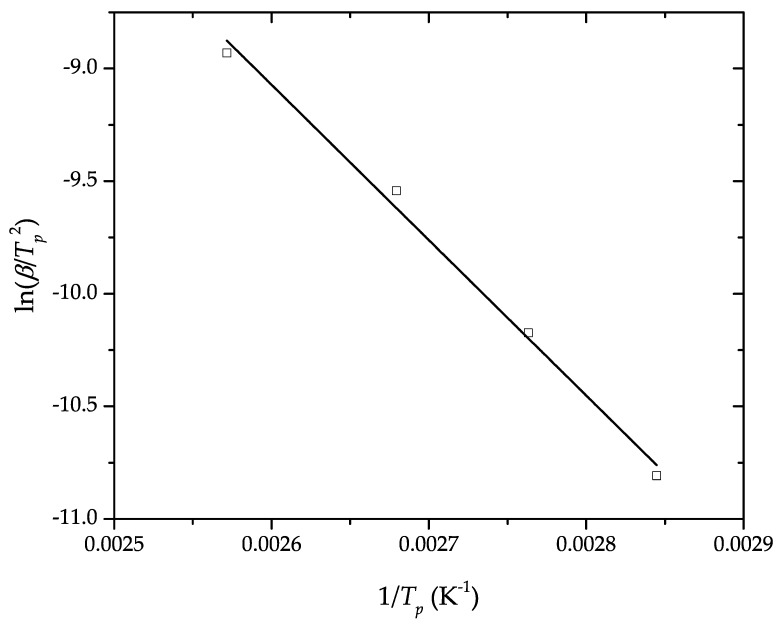
Plot of the experimental data according to the Kissinger method and the linear fitting according to Equation (15) corresponding to the crosslinking of a partially bio-based epoxy resin for different heating rates, β.

**Figure 6:**
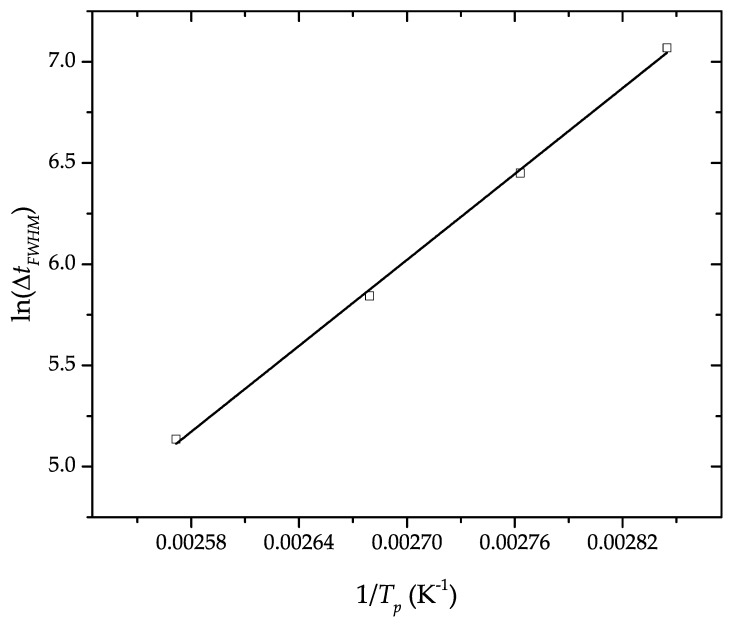
Plot of ln Δ*t_FWHM_* vs 1/*T_p_* to check the assumptions of the Kissinger method corresponding to the crosslinking of a partially bio-based epoxy resin for different heating rates, β.

One of the most important parameters of a kinetic study is the apparent activation energy, *E_a_*. As indicated previously, the Kissinger method allows for estimating a single value of *E_a_* for the curing process by using Equation (15). One important condition needed to apply the Kissinger method is that the conversion at the maximum reaction rate, denoted as $\alpha_{p}$, must be very similar for all the heating rates. As can be observed in Table 3, $\alpha_{p}$ showed an average value of 0.41 ± 0.01, and all the values corresponding to different heating rates are quite similar. Therefore, in a first instance, the Kissinger method could be applied. Figure 5 shows the typical plot representation of the Kissinger method, showing good linear fitting. In particular, the *E_a_* obtained from the slope was 57.3 ± 3.1 kJ mol^−1^. As it has been indicated previously, the peak width of the curing rate is also representative for a single step process. For this reason, a plot of ln Δ*t_FWHM_* versus 1/*T_p_* has been used to check this. The experimental data and the corresponding linear fit can be seen in Figure 6. The *E_a_* value obtained from the peak width at half maximum was 57.4 ± 3.1 kJ mol^−1^. This value is in good agreement with that obtained using the Kissinger method, thus suggesting the curing process took place in a single step process. The reactions that take place during a polymerization process could be quite complex due to the polymer chemical structure, diffusion phenomena, crosslinking density, monomer functionality, and chain mobility, among others [50]. Despite this, the overall process for this particular system, analysed using differential scanning calorimetry, appears to be a single step process (quite homogeneous and symmetric exothermic peak). Therefore, although there could be different activation energies for different processes occurring in the crosslinking, by using DSC, it is possible to obtain a unique “apparent” activation energy, *E_a_*, representative for all the processes that are overlapped.

**Figure 7:**
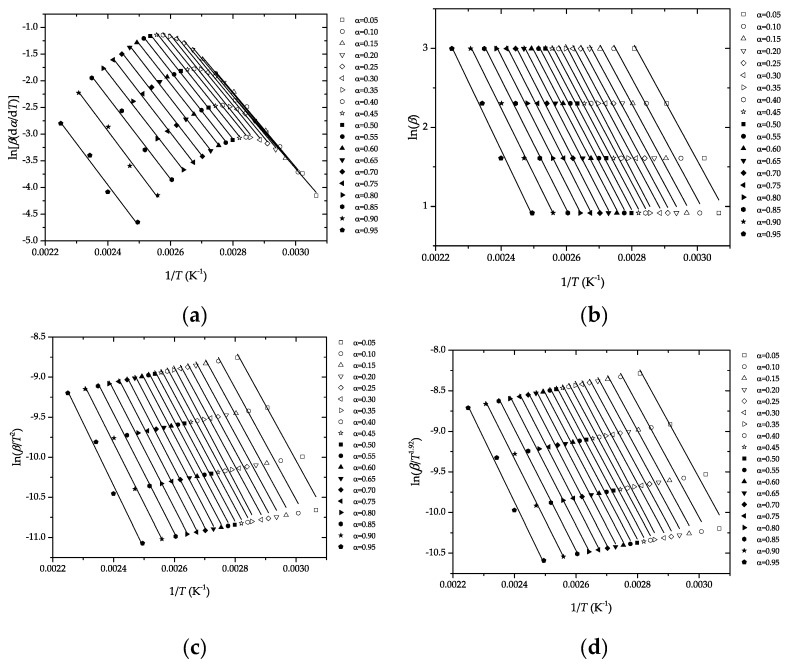
Characteristic plots of different isoconversional kinetic methods at different conversions α, corresponding to the crosslinking of a partially bio-based epoxy resin for different heating rates, β: (**a**) Friedman, (**b**) Flynn–Wall–Ozawa (FWO), (**c**) Kissinger–Akahira–Sunose (KAS), and (**d**) Starink.

Although the Kissinger method is easy to use and gives an idea of the apparent activation energy of the curing process, it gives a single *E_a_* value. Therefore, the possible change of *E_a_* with the conversion cannot be evaluated using the Kissinger method. To this end, several isoconversional methods have been used. Both differential and integral methods have been used and the corresponding plots are gathered in Figure 7. The Friedman method (Figure 7a) shows the calculated data at different conversions and several heating rates. As indicated by Equation (10), the *E_a_* can be obtained through the slope of the linear fits. The Pearson’s correlation coefficient *r* for all the linear fits at different conversions was about −0.995. The average *E_a_* obtained with the Friedman method was 61.2 ± 2.6 kJ mol^−1^. This value is within the same range of the *E_a_* obtained using the Kissinger method. In a first approach, the Friedman method did not use any approximation, so it should be more accurate than integral methods since they use different approximations of the temperature integral. However, due to a noisy signal, errors related to the baseline of DSC curves, and so on, there is no great difference between the Friedman method and other integral methods [43]. The Flynn–Wall–Ozawa (FWO) method is an integral method that uses a quite crude approximation of the temperature integral. Nevertheless, it is widely used and gives quite accurate values of *E_a_* as seen in Figure 7b. After applying Equation (12), the average *E_a_* obtained using the FWO method was 62.8 ± 3.1 kJ mol^−1^ with excellent correlation coefficients of *r* ≈ −0.995. The low standard deviation was representative of a slight change in *E_a_* with the conversion. A more accurate approximation of the temperature integral is used by the Kissinger–Akahira–Sunose (KAS) method (see Figure 7c). The correlation factor was also close to −0.995 for all linear fits. The average *E_a_* obtained using the KAS method was 59.8 ± 3.3 kJ mol^−1^, which is similar to all the previously reported values. The Starink method is an integral method that uses a very accurate approximation of the temperature integral and gave *r* values of about −0.995 (Figure 5d). By using the linear fits, the average *E_a_* was 60.0 ± 3.3 kJ mol^−1^.

**Figure 8:**
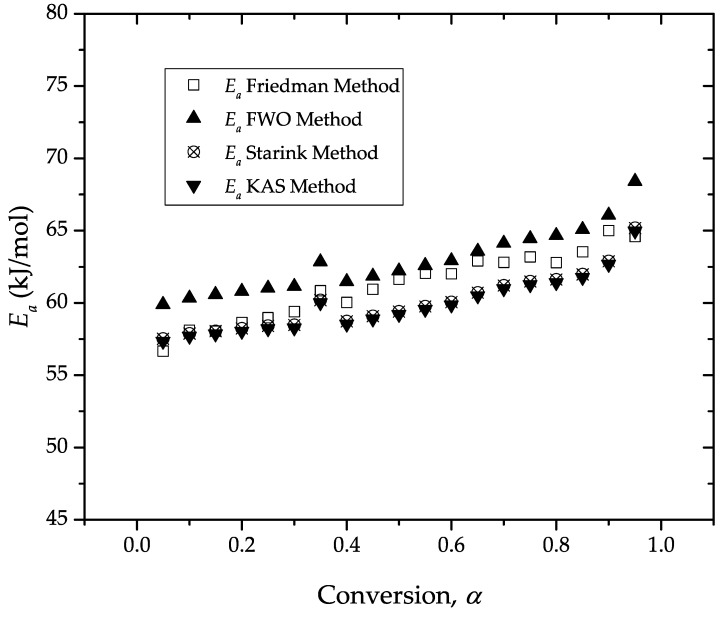
Variation of the apparent activation energy, *E_a_*, as a function of function of conversion α, corresponding to the crosslinking of a partially bio-based epoxy resin for different heating rates.

All the above-mentioned methods give an apparent activation energy between 57 and 62 kJ·mol^−1^, which is in total accordance with typical *E_a_* values for DGEBA-based epoxy resins, thus suggesting the plant-derived reactive diluent did not affect in a remarkable way the curing process α of the resin [11]. As suggested by the standard deviation of *E_a_* from different methods, there is very low variation of *E_a_* with α, as can be seen in Figure 8.

**Figure 9:**
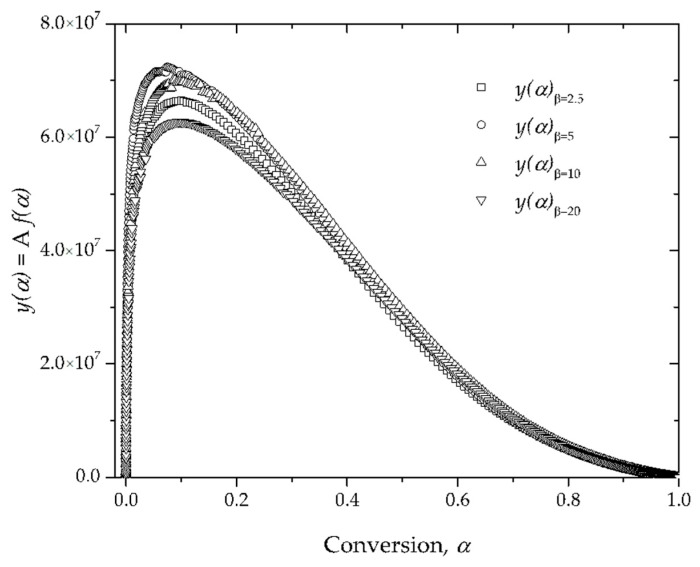
Calculated y(α) plots as a function of the conversion α, corresponding to the crosslinking of a partially bio-based epoxy resin for different heating rates.

Due to this low variability of *E_a_*, it can be considered that the curing process of this partially bio-based epoxy resin follows a single step process with a global “apparent” activation energy defined by the average for all the conversion range [57]. Once the *E_a_* has been accurately obtained, it is important to obtain the remaining kinetic parameters to complete the kinetic triplet. This means obtaining the reaction model f(α), and the pre-exponential factor, *A*. As indicated previously, Málek and Criado-Maqueda described a methodology to compare the calculated y(α) and z(α), as indicated by Equation (16) and Equation (17), respectively, with different master plots typical of thermally activated processes, as summarized in Table 1. Figure 9 shows the plot of y(α) as a function of the conversion α for different heating rates. As it can be seen, the maximum of the y(α), denoted as $\alpha_{M}$, was not zero ($\alpha_{M}$ = 0 could be representative for an *n*th order reaction model), thus suggesting another reaction model. In fact, this maximum was located close to 0.1 and the typical geometry suggested an autocatalytic reaction model as obtained by comparison with different master plots [51,55,58]. The maximum of the calculated y(α) functions for different heating rates are summarized in Table 4.

**Figure 10:**
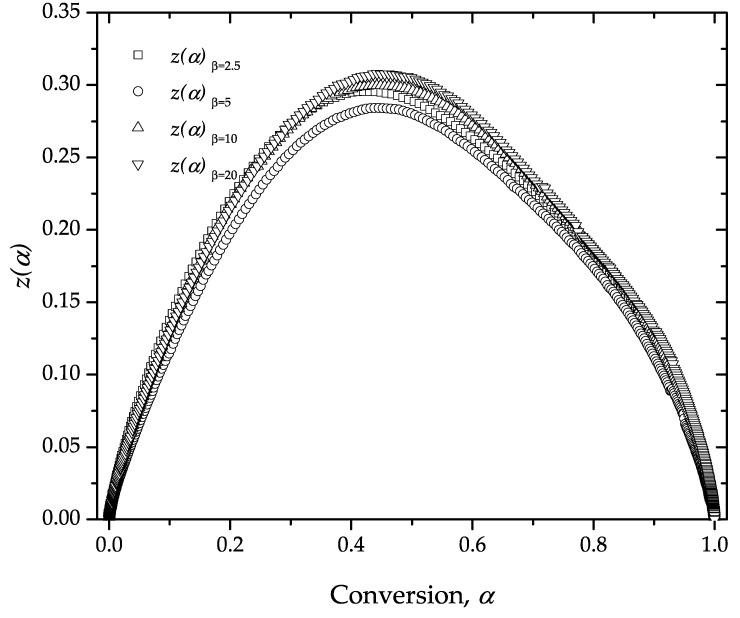
Calculated z(α) plots as a function of the conversion α, corresponding to the crosslinking of a partially bio-based epoxy resin for different heating rates.

Figure 10 gathers the z(α) plots for different heating rates. As can be seen, the z(α) functions show the same geometry, and some of them are overlapped. In can be clearly seen that the maximum of the z(α) function, denoted as $\alpha_{p}^{\infty }$, was between 0.4 and 0.5. The actual values for the different heating rates were obtained from the experimental values of z(α) and are shown in Table 4. By comparison of the experimental z(α) plots with some generalized master plots [58], this particular geometry is consistent with an autocatalytic model with a reasonable weight of the autocatalytic effect.

The peak for y(α) functions, $\alpha_{M}$, was 0.090 ± 0.011, and this means there was a certain autocatalytic effect on the curing reaction [59,60]. In fact, one of the conditions for an autocatalytic process requires $\alpha_{M}$ > 0. The autocatalytic effect will be more intense with higher $\alpha_{M}$ values. With regard to the maximum of the z(α) plots, the average $\alpha_{p}^{\infty }$ was 0.436 ± 0.011. Another condition for an autocatalytic process is that $\alpha_{M}$ < $\alpha_{p}^{\infty }$, and obviously, this is true. Therefore, it is possible to use an autocatalytic reaction model to obtain the remaining parameters for the kinetic triplet. For the curing of epoxies, the two parameters Šesták–Berggren reaction model [SB(*m*,*n*)] [51] can be used as suggested by both y(α) and z(α) functions. The Šesták–Berggren function is shown in Equation (25) and considers two parameters, *n* and *m*. The *n* parameter represents the typical *n*th order reaction models as it indicates the reaction rate is proportional to the unreacted material (1 − α), while the *m* exponent is represents the autocatalytic effect as it indicates, the conversion rate is proportional to the reacted material, α:

(25) $$f(\alpha )=\alpha^{m}{\left(1-\alpha \right)}^{n}$$

Once the reaction model has been checked, Equation (25) can be substituted into Equation (3), thus leading to Equation (26):

(26) $$\frac{d\alpha }{dt}=A\;e^{\frac{{-E}_{a}}{RT}}\;\alpha^{m}{\left(1-\alpha \right)}^{n}$$

As the apparent activation energy is known (isoconversional methods), it is possible to write Equation (26) as:

(27) $$\frac{\left(\frac{d\alpha }{dt}\right)}{e^{\frac{{-E}_{a}}{RT}}}=A\;\alpha^{m}{\left(1-\alpha \right)}^{n}=\frac{\left(\beta \frac{d\alpha }{dT}\right)}{e^{\frac{{-E}_{a}}{RT}}}$$

It is possible to obtain the *n* and *m* exponents using an iterative linear fit, but in this work, a non-linear curve fitting has been used. By applying natural logarithms to both sides of Equation (27), we obtain:

(28) $$\ln\left(\beta \frac{d\alpha }{dT}\right)+\frac{E_{a}}{RT}=\ln(A)+m\ln\alpha +n\ln(1-\alpha )$$

The term on the left-hand side of Equation (28) is ln(y(α)), such that a non-linear curve fitting, as indicated in Equation (29), can be used to obtain the optimum *m* and *n* exponents, as well as the pre-exponential factor, *A*:

(29) y=k+mln(x)+nln(1−x)

The parameters obtained after the non-linear curve fitting are summarized in Table 5. The average value of ln(*A*) was 18.47 ± 0.05, and regarding the reaction model exponents, *m* = 0.15 ± 0.01 and *n* = 1.76 ± 0.08. The low standard deviation indicates these values did not change in a remarkable way with the heating rate.

For an autocatalytic reaction model, the maximum of y(α) is related to the *n* and *m* exponents via Equation (30) [51,61]:

(30) $$\alpha_{M}=\frac{m}{m+n}$$

Substitution of the average *n* and *m* values obtained with the non-linear curve fitting led to a theoretical $\alpha_{M}$ of about 0.08, which is in total accordance with the calculated value from experimental data.

**Figure 11:**
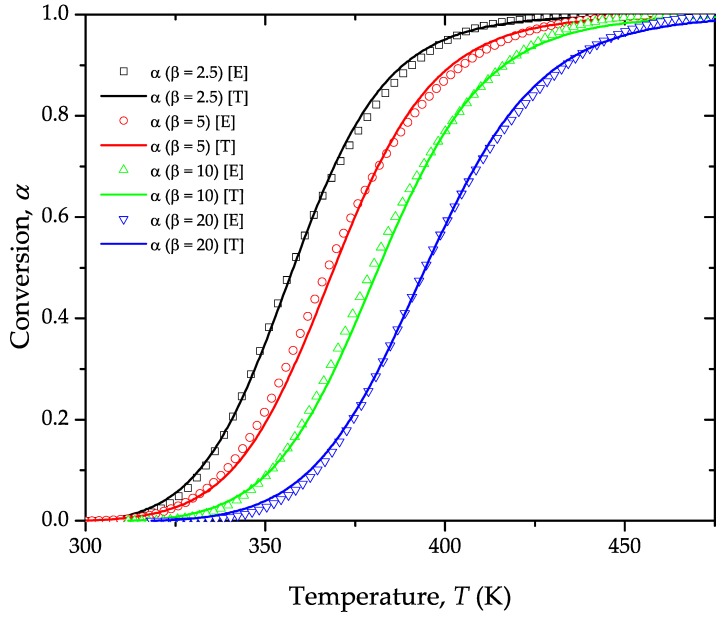
Comparison of conversion, α, corresponding to the crosslinking of a partially bio-based epoxy resin for different heating rates. Symbols represent experimental values [E] and lines represent theoretical [T] values.

Once the kinetic triple had been determined, the model wa integrated. Figure 11 shows a comparison of the experimental data (symbols) and the corresponding theoretical models (lines). As it can be seen, Equation (3), with the obtained kinetic triplet (*E_a_*, *A*, and f(α)), gave good accuracy relative to the experimental data for all the heating rates, thus giving consistency to the obtained parameters.

## 5. Conclusions

The cure kinetics of a partially bio-based epoxy resin derived from DGEBA and a plant-based reactive diluent (representing 31% of the cured resin) was analyzed using dynamic DSC at different heating rates. The apparent activation energy, *E_a_*, was determined using different isoconversional methods, and it was found to be between 57 and 62 kJ mol^−1^. The low dispersion of *E_a_* values for different conversion, α, values suggested a relatively independence of *E_a_* from α.

The reaction model was obtained via comparison of the calculated y(α) and z(α) with some well-established reaction models of thermally activated processes. It was concluded that the two-parameter [SB(*m*,*n*)] autocatalytic kinetic model of Šesták–Berggren was the most suitable for the description of the curing process of this partially bio-based epoxy resin. Therefore, this work provides a methodology to obtain the kinetic triplet corresponding to the curing/crosslinking of an epoxy resin. The theoretical curves show a great agreement with the experimental data, thus giving consistency to the obtained kinetic triplet.

## Figures and Tables

**Table 1:** Summary of some f(α) and g(α) functions corresponding to crosslinking of polymers.

Reaction Model		*f*(*α*)	*g*(*α*)
P2/3	Power law	2/3*α*^−1/2^	*α* ^3/2^
P2	Power law	2*α*^1/2^	*α* ^1/2^
A_2_	Avrami–Erofeev	2(1 − *α*)[−ln(1 − *α*)]^1/2^	[−ln(1 − *α*)]^1/2^
D_1_	Diffusion in one dimension	1/2*α*^−1^	*α* ^2^
D_2_	Valensi equation	−[ln(1 − *α*)]^−1^	(1 − *α*) ln (1 − *α*) + *α*
F_1_	Mample first order.	(1 − *α*)	−ln(1 − *α*)
F_2_	Random nucleation with two nuclei of individual particle	(1 − *α*)^2^	(1 − *α*)^−1^
F*_n_*	*n*-order reaction	(1 − *α*)^n^	[(1 − *α*)^1 − n^ − 1]/(n − 1)

**Table 2:** Summary of some physical properties of the epoxy system based on Resoltech^®^ 1070 partially bio-based epoxy resin and Resoltech^®^ 1074 hardener.

Property	Resoltech^®^ 1070	Resoltech^®^ 1074	Uncured Mixture (100:35)
Density at 23 °C (g cm^−3^)	1.18	0.96	1.22
Viscosity at 23 °C (mPa·s)	1750	50	700

**Table 3:** Parameters corresponding to DSC characterization of a partially bio-based epoxy system at different heating rates. First heating cycle: DSC characterization of the curing/crosslinking of the liquid resin, and second heating cycle: DSC characterization of the cured resin.

Heating Rate β (K min^−1^)	First Heating Cycle			Second Heating Cycle
	*T_p_* (K)	$\alpha_{p}$	$\Delta H_{T}\;(J\;g^{-1})$	*T_g_* (K)
2.5	351.5	0.401	373.9	368.8
5	361.9	0.425	366.5	367.7
10	373.2	0.408	305.5	365.5
20	388.9	0.417	285.8	364.1

**Table 4:** Maximum values of the calculated y(α) and z(α) functions corresponding to the crosslinking of a partially bio-based epoxy resin for different heating rates.

Heating Rate β (K min^−1^)	$\alpha_{M}$	$\alpha_{p}^{\infty }$
2.5	0.099	0.420
5	0.076	0.438
10	0.085	0.442
20	0.098	0.443

**Table 5:** Calculated kinetic parameters for autocatalytic SB(*m*,*n*) model by using a non-linear curve fitting, corresponding to the crosslinking of a partially bio-based epoxy resin for different heating rates.

Heating Rate β (K min^−1^)	ln (*A*) (*A* in min^−1^)	*m*	*n*
2.5	18.464	0.156	1.792
5	18.546	0.143	1.866
10	18.460	0.148	1.677
20	18.415	0.155	1.712

## References

[1] Jin F.L., Li X., Park S.J. (2015). Synthesis and application of epoxy resins: A review. J. Ind. Eng. Chem..

[2] Ratna D., Patri M., Chakraborty B.C., Deb P.C. (1997). Amine-Terminated Polysulfone as Modifier for Epoxy Resin. J. Appl. Polym. Sci..

[3] David R., Raja V.S., Singh S.K., Gore P. (2018). Development of anti-corrosive paint with improved toughness using carboxyl terminated modified epoxy resin. Prog. Org. Coat..

[4] Minty R.F., Thomason J.L., Yang L., Stanley W., Roy A. (2019). Development and application of novel technique for characterising the cure shrinkage of epoxy resins. Polym. Test..

[5] François C., Pourchet S., Boni G., Rautiainen S., Samec J., Fournier L., Robert C., Thomas C.M., Fontaine S., Gaillard Y. (2017). Design and synthesis of biobased epoxy thermosets from biorenewable resources. C. R. Chim..

[6] Zabihi O., Ahmadi M., Nikafshar S., Chandrakumar Preyeswary K., Naebe M. (2018). A technical review on epoxy-clay nanocomposites: Structure, properties, and their applications in fiber reinforced composites. Compos. Part B Eng..

[7] Jin H., Miller G.M., Sottos N.R., White S.R. (2011). Fracture and fatigue response of a self-healing epoxy adhesive. Polymer.

[8] Jin H., Miller G.M., Pety S.J., Griffin A.S., Stradley D.S., Roach D., Sottos N.R., White S.R. (2013). Fracture behavior of a self-healing, toughened epoxy adhesive. Int. J. Adhes. Adhes..

[9] Ramezanzadeh B., Niroumandrad S., Ahmadi A., Mahdavian M., Mohamadzadeh Moghadam M.H. (2016). Enhancement of barrier and corrosion protection performance of an epoxy coating through wet transfer of amino functionalized graphene oxide. Corros. Sci..

[10] Zhang S.Y., Ding Y.F., Li S.J., Luo X.W., Zhou W.F. (2002). Effect of polymeric structure on the corrosion protection of epoxy coatings. Corros. Sci..

[11] Yi C., Rostron P., Vahdati N., Gunister E., Alfantazi A. (2018). Curing kinetics and mechanical properties of epoxy based coatings: The influence of added solvent. Prog. Org. Coat..

[12] Zhao X., Li Y., Chen W., Li S., Zhao Y., Du S. (2019). Improved fracture toughness of epoxy resin reinforced with polyamide 6/graphene oxide nanocomposites prepared via in situ polymerization. Compos. Sci. Technol..

[13] Ho T.H., Wang C.S. (1996). Modification of epoxy resins with polysiloxane thermoplastic polyurethane for electronic encapsulation: 1. Polymer.

[14] Loccufier E., Geltmeyer J., Daelemans L., D’Hooge D.R., De Buysser K., De Clerck K. (2018). Silica Nanofibrous Membranes for the Separation of Heterogeneous Azeotropes. Adv. Funct. Mater..

[15] Riemenschneider W., Bolt H.M. (2005). Esters, Organic, Ullmann’s Encyclopedia of Industrial Chemistry.

[16] Dell’Erba I.E., Williams R.J.J. (2006). Homopolymerization of epoxy monomers initiated by 4-(dimethylamino)pyridine. Polym. Eng. Sci..

[17] Francis B., Lakshmana Rao V., Jose S., Catherine B.K., Ramaswamy R., Jose J., Thomas S. (2006). Poly(ether ether ketone) with pendent methyl groups as a toughening agent for amine cured DGEBA epoxy resin. J. Mater. Sci..

[18] Li L., Yu Y., Wu Q., Zhan G., Li S. (2009). Effect of chemical structure on the water sorption of amine-cured epoxy resins. Corros. Sci..

[19] Bell J.P., Carolina N.P. (1970). Structure of a typical amine-cured epoxy resin. Polymer.

[20] Ellis B., Ashcroft W.R., Shaw S.J., Cantwell W.J., Kausch H.H., Johari G.P., Jones F.R., Chen X.M. (1993). Chemistry and Technology of Epoxy Resins.

[21] Bertomeu D., García-Sanoguera D., Fenollar O., Boronat T., Balart R. (2012). Use of Eco-Friendly Epoxy Resins From Renewable Resources as Potential Substitutes of Petrochemical Epoxy Resins for Ambient Cured Composites with Flax Reinforcements. Polym. Compos..

[22] Samper M.D., Petrucci R., Sánchez-Nacher L., Balart R., Kenny J.M. (2015). New environmentally friendly composite laminates with epoxidized linseed oil (ELO) and slate fiber fabrics. Compos. Part B Eng..

[23] Samper M.D., Petrucci R., Sanchez-Nacher L., Balart R., Kenny J.M. (2015). Properties of composite laminates based on basalt fibers with epoxidized vegetable oils. Mater. Des..

[24] España J.M., Sánchez-Nacher L., Boronat T., Fombuena V., Balart R. (2012). Properties of biobased epoxy resins from epoxidized soybean oil (ESBO) cured with maleic anhydride (MA). JAOCS J. Am. Oil Chem. Soc..

[25] Samper M.D., Petrucci R., Sánchez-Nacher L., Balart R., Kenny J.M. (2015). Effect of silane coupling agents on basalt fiber-epoxidized vegetable oil matrix composite materials analyzed by the single fiber fragmentation technique. Polym. Compos..

[26] Raquez J.M., Deléglise M., Lacrampe M.F., Krawczak P. (2010). Thermosetting (bio)materials derived from renewable resources: A critical review. Prog. Polym. Sci. (Oxf.).

[27] Meier M.A.R., Metzger J.O., Schubert U.S. (2007). Plant oil renewable resources as green alternatives in polymer science. Chem. Soc. Rev..

[28] Stemmelen M., Pessel F., Lapinte V., Caillol S., Habas J.P., Robin J.J. (2011). A fully biobased epoxy resin from vegetable oils: From the synthesis of the precursors by thiol-ene reaction to the study of the final material. J. Polym. Sci. Part A Polym. Chem..

[29] Michałowicz J. (2014). Bisphenol A—Sources, toxicity and biotransformation. Environ. Toxicol. Pharmacol..

[30] Carbonell-Verdu A., Bernardi L., Garcia-Garcia D., Sanchez-Nacher L., Balart R. (2015). Development of environmentally friendly composite matrices from epoxidized cottonseed oilv. Eur. Polym. J..

[31] Ortiz R.A., López D.P., Cisneros M.D.L.G., Valverde J.C.R., Crivello J.V. (2005). A kinetic study of the acceleration effect of substituted benzyl alcohols on the cationic photopolymerization rate of epoxidized natural oils. Polymer.

[32] Biermann U., Friedt W., Lang S. (2000). New syntheses with oils and fats as renewable raw materials for the chemical industry. Angew. Chem..

[33] Khot S.N., Lascala J.J., Can E., Morye S.S., Williams G.I., Palmese G.R., Kusefoglu S.H., Wool R.P. (2001). Development and application of triglyceride-based polymers and composites. J. Appl. Polym. Sci..

[34] Ferri J.M., Samper M.D., García-Sanoguera D., Reig M.J., Fenollar O., Balart R. (2016). Plasticizing effect of biobased epoxidized fatty acid esters on mechanical and thermal properties of poly(lactic acid). J. Mater. Sci..

[35] Carbonell-Verdu A., Garcia-Sanoguera D., Jordá-Vilaplana A., Sanchez-Nacher L., Balart R. (2016). A new biobased plasticizer for poly(vinyl chloride) based on epoxidized cottonseed oil. J. Appl. Polym. Sci..

[36] Fenollar O., Garcia-Sanoguera D., Sanchez-Nacher L., Lopez J., Balart R. (2010). Effect of the epoxidized linseed oil concentration as natural plasticizer in vinyl plastisols. J. Mater. Sci..

[37] Fenollar O., Garcia-Sanoguera D., Sánchez-Nácher L., López J., Balart R. (2012). Characterization of the curing process of vinyl plastisols with epoxidized linseed oil as a natural-based plasticizer. J. Appl. Polym. Sci..

[38] Samper M.D., Fombuena V., Boronat T., García-Sanoguera D., Balart R. (2012). Thermal and mechanical characterization of epoxy resins (ELO and ESO) cured with anhydrides. JAOCS J. Am. Oil Chem. Soc..

[39] Park S.J., Jin F.L., Lee J.R. (2004). Effect of biodegradable epoxidized castor oil on physicochemical and mechanical properties of epoxy resins. Macromol. Chem. Phys..

[40] Pawar M., Kadam A., Yemul O., Thamke V., Kodam K. (2016). Biodegradable bioepoxy resins based on epoxidized natural oil (cottonseed & algae) cured with citric and tartaric acids through solution polymerization: A renewable approach. Ind. Crop. Prod..

[41] Incerti D., Wang T., Carolan D., Fergusson A. (2018). Curing rate effects on the toughness of epoxy polymers. Polymer.

[42] Javdanitehran M., Berg D.C., Duemichen E., Ziegmann G. (2016). An iterative approach for isothermal curing kinetics modelling of an epoxy resin system. Thermochim. Acta.

[43] Vyazovkin S., Burnham A.K., Criado J.M., Pérez-Maqueda L.A., Popescu C., Sbirrazzuoli N. (2011). ICTAC Kinetics Committee recommendations for performing kinetic computations on thermal analysis data. Thermochim. Acta.

[44] Friedman H.L. (2007). Kinetics of thermal degradation of char-forming plastics from thermogravimetry. Application to a phenolic plastic. J. Polym. Sci. Part C Polym. Symp..

[45] Doyle C.D. (1962). Estimating isothermal life from thermogravimetric data. J. Appl. Polym. Sci..

[46] Akahira T., Sunose T. (1969). Transactions of Joint Convention of Four Electrical Institutes.

[47] Starink M.J. (2003). The determination of activation energy from linear heating rate experiments: A comparison of the accuracy of isoconversion methods. Thermochim. Acta.

[48] Kissinger H.E. (1956). Variation of peak temperature with heating rate in differential thermal analysis. J. Res. Natl. Bur. Stand..

[49] Farjas J., Butchosa N., Roura P. (2010). A simple kinetic method for the determination of the reaction model from non-isothermal experiments. J. Therm. Anal. Calorim..

[50] D’hooge D.R., Van Steenberge P.H.M., Reyniers M.-F., Marin G.B. (2016). The strength of multi-scale modeling to unveil the complexity of radical polymerization. Prog. Polym. Sci..

[51] Málek J. (1992). The kinetic analysis of non-isothermal data. Thermochim. Acta.

[52] Senum G.I., Yang R.T. (1977). Rational approximations of the integral of the Arrhenius function. J. Therm. Anal..

[53] Pérez-Maqueda L.A., Criado J.M. (2000). Accuracy of Senum and Yang’s approximations to the Arrhenius integral. J. Therm. Anal. Calorim..

[54] Flynn J.H. (1997). The “temperature integral”—Its use and abuse. Thermochim. Acta.

[55] Vyazovkin S., Chrissafis K., Di Lorenzo M.L., Koga N., Pijolat M., Roduit B., Sbirrazzuoli N., Suñol J.J. (2014). ICTAC Kinetics Committee recommendations for collecting experimental thermal analysis data for kinetic computations. Thermochim. Acta.

[56] Hale A., Macosko C.W., Bair H.E. (1991). Glass Transition Temperature as a Function of Conversion in Thermosetting Polymers. Macromolecules.

[57] Vyazovkin S., Sbirrazzuoli N. (2006). Isoconversional kinetic analysis of thermally stimulated processes in polymers. Macromol. Rapid Commun..

[58] Criado J.M., Malek J., Ortega A. (1989). Applicability of the Master Plots in Kinetic Analysis. Thermochim. Acta.

[59] Bilyeu B., Brostow W., Menard K.P.K.P. (2001). Epoxy thermosets and their applications. III. Kinetic equations and models. J. Mater. Educ..

[60] Benedetti A., Fernandes P., Granja J.L., Sena-Cruz J., Azenha M. (2016). Influence of temperature on the curing of an epoxy adhesive and its influence on bond behaviour of NSM-CFRP systems. Compos. Part B Eng..

[61] Málek J. (1989). A computer program for kinetic analysis of non-isothermal thermoanalytical data. Thermochim. Acta.

